# Recent Advances in Electrospun Membranes for Radiative Cooling

**DOI:** 10.3390/ma16103677

**Published:** 2023-05-11

**Authors:** Dongxue Zhang, Haiyan Zhang, Zhiguang Xu, Yan Zhao

**Affiliations:** 1 College of Textile and Clothing Engineering, Soochow University, Suzhou 215123, China; 20204215025@stu.suda.edu.cn (D.Z.); 20204015004@stu.suda.edu.cn (H.Z.); 2 China-Australia Institute for Advanced Materials and Manufacturing, Jiaxing University, Jiaxing 314001, China

**Keywords:** radiative cooling, electrospun membrane, electrospinning, thermal management

## Abstract

Radiative cooling is an approach that maximizes the thermal emission through the atmospheric window in order to dissipate heat, while minimizing the absorption of incoming atmospheric radiation, to realize a net cooling effect without consuming energy. Electrospun membranes are made of ultra-thin fibers with high porosity and surface area, which makes them suitable for radiative cooling applications. Many studies have investigated the use of electrospun membranes for radiative cooling, but a comprehensive review that summarizes the research progress in this area is still lacking. In this review, we first summarize the basic principles of radiative cooling and its significance in achieving sustainable cooling. We then introduce the concept of radiative cooling of electrospun membranes and discuss the selection criteria for materials. Furthermore, we examine recent advancements in the structural design of electrospun membranes for improved cooling performance, including optimization of geometric parameters, incorporation of highly reflective nanoparticles, and designing multilayer structure. Additionally, we discuss dual-mode temperature regulation, which aims to adapt to a wider range of temperature conditions. Finally, we provide perspectives for the development of electrospun membranes for efficient radiative cooling. This review will provide a valuable resource for researchers working in the field of radiative cooling, as well as for engineers and designers interested in commercializing and developing new applications for these materials.

## 1. Introduction

Maintaining a comfortable living environment is a crucial aspect of modern life, but it comes at a significant cost to our planet. The energy used for effective cooling and heating accounts for about 32–33% of the global energy use [1,2,3]. With the rise of global temperatures due to climate change, the need for cooling is becoming even more critical. At present, space cooling is widely used in summer, mainly relying on traditional vapor compression-based technologies, which are energy-intensive and take up 17% of the world’s total electricity. Existing cooling methods have been leading to environmental issues including ozone depletion, the greenhouse effect, and air pollution. Research conducted by the Intergovernmental Panel on Climate change (IPCC) indicates that the global surface temperature has increased by 0.74 ± 0.18 °C over the past century [4,5]. Given these reasons, it is imperative to develop alternative cooling strategies, methods, and technologies.

Radiative cooling technology reflects solar irradiance and radiates heat through the “atmospheric window” to achieve cooling [6,7,8]. Unlike most cooling methods that need energy input to remove heat, radiative cooling is a way to achieve passive cooling without consuming energy [9,10,11,12,13]. The concept was first proposed by Arago in 1828 [14,15], and has since attracted attention from scientists worldwide. Radiative cooling can help reduce energy and environmental problems by saving energy and electricity [5,16,17,18,19]. For instance, for personal thermal management, wearing radiative cooling clothing can help individuals maintain their personal thermal comfort at higher ambient temperatures, reducing the need for air conditioning and saving energy in the process. By allowing for cooling setpoints of air conditioners to be higher while still maintaining the same level of personal thermal comfort, radiative cooling clothing can lead to significant energy savings. In fact, studies have shown that with an increase of 1–4 °C in the setpoint temperature, an energy saving of 7–45% can be achieved, depending on the specific conditions [20].

So far, radiative cooling materials have proven to be highly versatile and have been developed for a variety of applications including energy-efficient buildings [21,22,23,24,25,26], personal thermal management [20,27,28,29,30], cooling solar cells [31,32,33,34], enhancing dew yield in water harvesting [35,36], and generating temperature differential for thermoelectric generators [37,38,39,40]. In general, to achieve a cooling effect that is lower than the ambient temperature, even for the case under direct sunlight, radiative cooling materials need to possess two key characteristics: strong emission through the atmospheric window and the ability to reflect most of the sunlight. There are various preparation methods available for radiative cooling materials, including vacuum evaporation [17,41], micro–nano processing technology [42,43], direct coating [44], electrospinning [28], and extrusion molding [27,45]. Among these methods, electrospinning is the only technology that can produce continuous ultra-fine fibers with diameters ranging from several micrometers to several hundreds of nanometers, by applying an appropriate electric field on the viscose polymeric fluid [46]. This technique is applicable to almost all soluble or fusible polymers. The fiber diameter, morphology, alignment, porosity, and surface area of electrospun fibers can be easily adjusted by optimizing a series of parameters including equipment configuration, spinning conditions, material ratio, and solvent type [47,48]. The specific wavelength scattering properties of fiber-shaped materials make them an ideal candidate for radiative cooling applications. As a result, electrospun membranes have been extensively researched in recent years due to their excellent radiative cooling capabilities, for various applications including personal thermal management [49,50,51,52,53,54,55,56], passive cooling of buildings [57,58,59], temperature control of electronic devices [60,61], cooling down food and agricultural products [62], and automobile sun protection clothing [63,64,65].

To date, radiative cooling has been widely studied and a number of literature reviews have been published on this topic, which covers a wide range of aspects related to the design, fabrication, and application of radiative cooling materials [3,15,66,67,68,69,70,71,72,73,74,75,76,77,78,79,80,81,82,83]. However, despite the extensive coverage of radiative cooling in the literature and a few review papers that cover fiber-based materials obtained by different spinning techniques for radiative cooling applications [84], there is still a lack of comprehensive reviews that specifically address the research progress of radiative cooling electrospun membranes. In this review, we first briefly summarize the fundamental principles of radiative cooling and its significance in achieving sustainable cooling. We then introduce the concept of radiative cooling of electrospun membranes and discuss the selection criteria for materials used in this process. Furthermore, we examine recent advancements in the structural design of electrospun membranes for improved cooling performance, including optimization of geometric parameters, incorporation of highly reflective nanoparticles, and designing multilayer structure. Additionally, we discuss dual-mode temperature regulation, which aims to adapt to a wider range of temperature conditions. Finally, we provide perspectives for the development of electrospun membranes for efficient radiative cooling.

## 2. Basic Principles of Radiative Cooling

Heat is a form of energy that always transfers spontaneously from a region of high temperature to another region of lower temperature. In fact, the outer space outside the Earth’s atmosphere can be considered as a huge natural cold storage, with its temperature close to absolute zero. From the point of view of thermal radiation, the outer space can be regarded as a blackbody with zero absolute temperature, and all objects with temperatures higher than absolute zero will emit heat to the outer space via infrared radiation [69], through which heat is released from objects on the Earth into the cold universe via thermal radiation [85].

**Figure 1:**
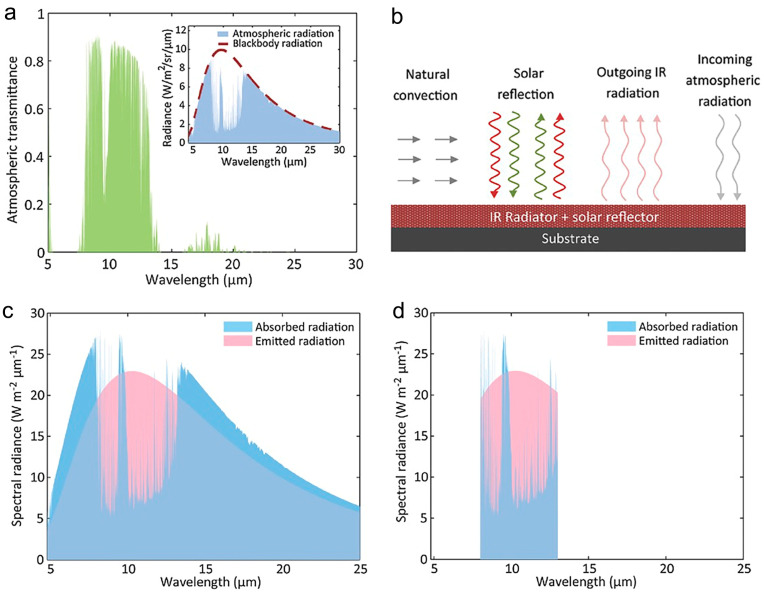
(**a**) A modeled atmospheric transmittance (Inset is the downward atmospheric radiation) [66]. (**b**) Schematics of the thermal exchange of an IR radiator with the environment through radiative and nonradiative processes [66]. Emitted radiation and absorbed radiation at 16 °C for (**c**) broadband and (**d**) selective radiators, respectively [66].

Although the radiative exothermic and endothermic processes of any object occur at the same time, the object can still cool down as long as the heat radiation is more than the heat absorbed from the atmosphere. This is because after receiving solar radiation on the Earth’s surface, the energy is mainly concentrated in the wavelength range of 2.5–50 μm to emit thermal radiation. Most of the energy is absorbed by the atmosphere, which is composed of ozone, water vapor, carbon dioxide, and suspended particles that absorb, scatter, and emit electromagnetic waves, where water vapor and carbon dioxide make the most contributions in the thermal radiation of the atmosphere [85]. On one hand, the atmosphere has a highly transparent window ranging from 8 μm to 13 μm (Figure 1a), where the atmosphere’s radiative emission is very weak [66]. Outside this window, the atmosphere is highly emissive. On the other hand, the peak thermal radiation of a black body defined by Planck’s law at around 300 K coincidentally falls within this atmospheric window. This feature allows for a terrestrial body to cool down by removing heat in the form of thermal emission through the atmospheric window, which is the passive radiative cooling mechanism.

For practical use, the cooling property of a radiator is highly dependent on factors including the incoming atmospheric radiation, the solar radiation during daytime, and the convective or conductive heat gain (Figure 1b). To achieve high cooling efficiency, the thermal emission through the atmospheric window must be maximized, while the incoming atmospheric radiation, the nonradiative heat gain from the surrounding and absorption of solar light must be minimized [66]. Moreover, the spectral emission profiles of the radiative materials significantly affect the performance of radiative cooling. There are two types of radiative materials. The first type is the broadband radiative materials, which are similar to blackbody and have emissivity within the entire emission wavelength range of the atmosphere (Figure 1c). The second type is the selective radiative materials that have high emissivity in 8–13 μm wavelength range, but very low emissivity outside the 8–13 μm window (Figure 1d). For broadband radiative materials, it is noted that they may receive more radiative power than they emit at the ambient temperature, which may prevent them from cooling well below the ambient temperature. In comparison, the selective radiative materials do not absorb radiative power outside the atmospheric window (while in the 8–13 μm window, the atmosphere is highly emissive), but strongly emit within the 8–13 μm wavelength range, which ensures that the nonradiative or solar heat gain can be overcome and thus it is possible to realize effective cooling effects below the ambient temperature. However, for applications where the aim is to decrease the temperature of a device producing a lot of heat, rather than to cool the device below the ambient temperature, the broadband materials can be more useful than the selective radiative materials.

## 3. Radiative Cooling of Electrospun Membranes

**Figure 2:**
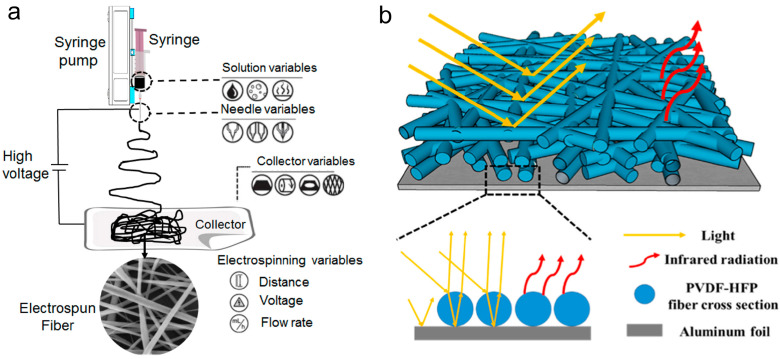
Schematic illustration of (**a**) the electrospinning process [86] and (**b**) the radiative cooling principle of electrospun membranes. Reprinted with permission from [60]. Copyright 2021 Elsevier.

Electrospinning is a well-established technique that enables the production of submicro- and nanofibers with great precision and control. In the electrospinning process (Figure 2a) [86], as the electric field is applied, the droplet at the tip of the needle elongates into a cone shape known as the Taylor cone. When the electric field exceeds the surface tension of the solution, a jet is formed, and the solvent evaporates as the jet travels from the needle tip to the collector, leading to the formation of an ultrafine fiber. The structure and morphology of the fibers can be adjusted by controlling the parameters of the electrospinning process, including the electric field strength, polymer concentration, solution viscosity, flow rate of the polymer solution, and the distance between the spinneret and collector. For instance, by increasing the polymer concentration, the diameter of the resulting fibers can be increased, while decreasing the concentration can produce smaller diameter fibers. By increasing the electric field strength, the fibers can be stretched, resulting in thinner fibers. Similarly, by changing the distance between the spinneret and collector, the fibers can be oriented in different directions, which can affect their mechanical and optical properties.

Electrospun membranes are a promising type of material for radiative cooling applications due to their high porosity, large surface area, and tunable optical properties. The large surface area allows for a greater amount of heat transfer through radiation, as more surface area is available for the material to emit radiation. Electrospun membranes can also be designed to have specific optical properties, such as high reflectivity to the solar light by adjusting fiber diameter and pore size, and high emissivity in the 8–13 μm window by selecting high emissivity materials. This allows them to reflect sunlight and emit thermal radiation efficiently (Figure 2b) [60], which further enhances their cooling performance. More importantly, the electrospun membranes are characterized by an in-plane anisotropic structure with fibers arranged mostly in a planar fashion. It has been confirmed that such an anisotropic structure provides higher diffuse reflectance when illuminated from the perpendicular direction than isotropic alignment [87].

In terms of other properties, electrospun membranes for radiative cooling need to be strong enough to maintain their shape and structure over time, without becoming brittle or losing their elasticity. In addition, thermal stability is also important for electrospun membranes to maintain their structural integrity and cooling performance even under high temperatures. This is crucial for applications in hot and sunny climates where radiative cooling can be most effective. Electrospun membranes also have the potential for reusability, as they can be easily maintained and reused in radiative cooling systems. However, the actual reusability of these membranes depends on various factors such as the type of polymer used and the specific conditions of their use.

For electrospun membranes used for radiative cooling, besides the general characterizations such as their mechanical, thermal, and wetting properties, spectroscopic characterization of their emissivity, reflectivity, transmissivity, and absorptivity at specific wavelength ranges is usually conducted. Some tests that are often specifically tailored for electrospun membranes, such as measuring the fiber diameter, the porosity, and the orientation degree of fibers, are also performed. Moreover, the radiative cooling performance can be directly evaluated by the thermal measurement of a radiative cooling surface. For the thermal measurement, the membrane is enclosed by insulation foam, and a convection shield is used to prevent non-radiative heat transfer (conduction and convection heat loss) between the ambient environment and the radiative cooling surface (at sub ambient temperature). Typically, materials with high transmittance within the atmospheric window, such as low-/high-density polyethylene, are used as the convection shield.

In addition, it is noted that the thermal properties of electrospun membranes play an important role in passive radiative cooling applications. Due to their porous structure, electrospun membranes usually exhibit quite low thermal conductivity [88,89], which is one of the main limitations of them when they are used to decrease the temperature of hot substrates, because they are not able to efficiently extract and dissipate excess heat from the substrates. However, for the case of sub ambient cooling, environmental heat gain significantly affects the cooling performance at sub ambient temperatures, and thus it is important to isolate the heat gain from the environment. Therefore, good thermal insulation against ambient air is advantageous for achieving sub ambient cooling. For instance, Zhong et al. [58] prepared a thermal insulating membrane consisting of coaxially electrospun hierarchically hollow microfibers. The membrane shows a high solar reflectivity of 94% and also a high infrared thermal emissivity of 0.94, while it has a lower thermal conductivity than air, which helps to effectively shield the ambient thermal gain. It is worth noting that the influence of the incorporation of thermal insulating function is negligible to the radiative cooling performance. Under direct sunlight radiation with an intensity of 900 W/m^2^, the membrane shows a temperature drop of about 9 °C.

In the following sections, the associated factors affecting the cooling performance of electrospun membranes, including the selection of materials and structural design, as well as the dual-mode temperature regulation, are discussed.

## 4. Selection of Materials

Based on the above discussion, it is obvious that the maximization of radiative emission through the 8–13 μm window and the minimization of heat gain (especially the solar radiation during the daytime) are two necessary approaches to achieving high cooling efficiency [66]. Therefore, to enhance radiative cooling, it would be useful to choose materials with high emissivity within the atmospheric window. For the case of cooling during the daytime, on the other hand, it is highly important to improve the reflectivity to solar light and make it cooperate with the high emissivity to achieve high cooling performance.

### 4.1. High Emissivity Materials

As discussed above, the radiative cooling performance is highly dependent on the emission profile of the two types of radiative cooling materials. Both types of radiative materials should have low absorptivity to the solar light. Regarding the cooling performance, the use of broadband materials is to maximize the net cooling power, while the use of selective ones is to maximize the temperature difference between the radiator and the environment [15]. Many materials inherently emit highly across the entire IR wavelength range and the emissivity is almost uniform, and thus most radiative materials belong to the type of broadband materials [15].

**Figure 3:**
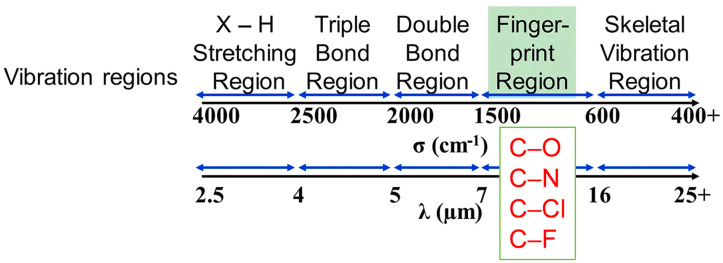
Vibration regions of functional groups in polymers. Reprinted with permission from [15]. Copyright 2021 Elsevier.

Polymers are commonly used as base materials for radiative cooling due to their process simplicity, low cost, and wide applicability. The IR emission of polymers depends on the vibrations of their functional groups [90]. As indicated in Figure 3, the fingerprint region (6.7–16.7 μm) corresponds to the atmospheric window and can be used as a main selection metric to identify highly efficient radiative cooling polymers [15]. In the fingerprint region, groups such as C-O, C-F, C-N, and C-Cl have relatively strong absorption due to the bending vibration, and thus polymers having these groups can be selected for high-efficient radiative cooling. For the preparation of radiative cooling electrospun membranes, polymers such as poly(vinylidene fluoride-co-hexafluoropropylene) (PVDF-HFP) [59,61,91], polyethylene oxide (PEO) [92], cellulose acetate (CA) [93,94], and polyvinylidene fluoride (PVDF) [54,64,65,95] have been widely used.

### 4.2. High Reflectivity Materials

It is known that most polymer films (with high IR emissivity) are transparent, and they do not reflect sunlight. To solve this problem, metal substrates or inorganic nanoparticles (Al_2_O_3_, SiO_2_, ZnO, etc.) are often added for reflection. Some of these materials can not only strongly reflect visible light but also have high IR emission. For instance, Al_2_O_3_ has almost no absorption in the visible-near-infrared wavelength range (0.4–2 μm), and so it is difficult to produce heat under direct solar radiation, but it has strong phonon polarization in the mid-infrared regime (8–20 μm) and thus good emissivity [96,97]. SiO_2_ is also transparent to visible light, while it is capable of effectively emitting infrared light [11,60]. This occurs because SiO_2_ has a high refractive index for infrared light, which causes the light to be trapped and absorbed within the material. ZnO also possesses a high refractive index of approximately *n* ≈ 2, and also exhibits virtually no absorption in the visible to mid-infrared wavelength range of 0.4–16 µm [98,99]. TiO_2_ exhibits hyperspectral reflectivity in the wavelength range of visible and near-infrared [100], but it has strong UV absorption and has been rarely used for radiative cooling applications [57].

## 5. Structural Design

According to the scattering theory established by Rayleigh [101] and Mie [102], materials can strongly scatter electromagnetic waves with wavelengths close to their size. The apertures in the micron to nanometer scale can reflect the energy of sunlight in the visible band, preventing an increase in object temperature. Additionally, these apertures can enhance the emissivity of polymers in the infrared window band, leading to improved radiative cooling performance. In this section, we summarize the approaches employed to enhance the radiative cooling performance through structural design on fibers themselves and the entire electrospun membranes, including the optimization of geometric parameters, the incorporation of highly reflective nanoparticles, and the design of multilayer structures.

### 5.1. Optimization of Geometric Parameters

By changing the process parameters, the diameter of the electrospun fibers can be facilely adjusted from a few nanometers to a few microns. The influence of nanofiber diameter on cooling efficiency has been studied by using both scattering theory and experiments. The results indicate that the diameter of the fibers plays a critical role in the effectiveness of radiative cooling. For instance, Li et al. [92] prepared a PEO electrospun membrane and used Mie theory to calculate the change in the scattering efficiency of PEO nanofibers with the fiber diameter across the solar spectrum. The results showed that nanofibers with diameter in the range of 500–1200 nm can strongly scatter sunlight, especially in the 0.3–1.2 μm wavelength range. The PEO electrospun membrane exhibits a high reflectivity of >96.3% to the sunlight (0.3–2.5 μm) and a relatively high emissivity of 78% in the 8–13 μm wavelength range.

**Figure 4:**
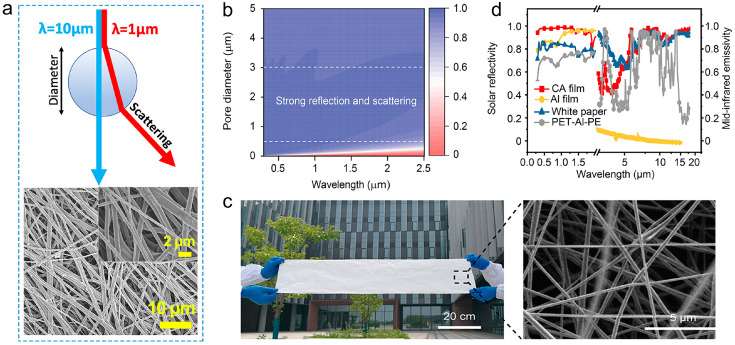
(**a**) Cross-sectional illustration of the optical scattering of different wavelengths across a fiber, and SEM image of PVDF fibers with an average diameter of 600 nm. Reprinted with permission from [95]. Copyright 2021 American Chemical Society. (**b**) Theoretical analysis of the effect of pore size on the scattering of the incident sunlight. On the right side of the graph, the color bar represents reflectivity [62]. (**c**) Digital photo and SEM image of the CA membrane [62]. (**d**) Mid-infrared emissivity and solar reflectivity spectra of the CA membrane [62].

Besides the high solar light reflectivity, high IR transmittance is important for the radiative heat dissipation of the human body. Kim et al. [95] optimized the fiber diameter for achieving high scattering at a shorter wavelength (~2500 nm), and also a relatively high transmittance at a longer wavelength (~20 μm), so as to simultaneously realize a high reflectivity to solar light (wavelength range 250–2500 nm) and a high transmittance to human body radiation (wavelength range 3–20 μm) (Figure 4a). They demonstrated that PVDF electrospun membrane with an average fiber diameter of ~600 nm has a remarkable radiative cooling ability with solar and NIR reflectance higher than 90%, to significantly block the sunlight energy influx, and an IR transmittance of ~50% for effective radiative heat dissipation of human body. On a simulated skin, the membrane shows a cooling effect of ~12 °C compared to normal textile materials.

In addition to the fiber diameter, the size of the pores in the electrospun membrane also affects the reflection of sunlight. Li et al. [62] theoretically analyzed the effect of the pore size on the scattering of the incident sunlight, revealing that a porous structure with multiscale pores having size in the range from 500 nm to 3 μm can strongly scatter and reflect the incident sunlight (300 nm to 2.5 μm wavelength range) (Figure 4b). According to this theoretical analysis, they prepared cellulose acetate (CA) electrospun membrane that has multiscale pores having sizes in the same range as that in the theoretical analysis (Figure 4c). It was found that the porous structure and the inherent molecular vibration of CA endow the membrane with a reflectivity as high as 0.974 for the solar spectrum (AM 1.5 G) and a broadband mid-infrared emissivity of 0.92 with an emissive peak within the atmospheric transparent window (Figure 4d). Under direct sunlight irradiation, the CA membrane achieved a cooling temperature of ~12 °C and a cooling power of ~110 W/m^2^. Cheng et al. [103] prepared hierarchically porous PVDF-HFP fibers with interconnected nanospheres structure, that possess large roughness and high specific surface area, via vapor-induced phase separation in the process of electrospinning. The PVDF-HFP membrane shows an average solar reflectance of ~93.7% and an IR emittance of ~91.9%, yielding a temperature drop of ~19.8 °C compared to the bare skin simulator under solar intensity of ~950 W/m^2^.

### 5.2. Incorporation of Highly Reflective Nanoparticles

According to Mie theory [102], dielectric and semiconductor nanoparticles with high-refractive index, in a specific size range, may exhibit strong resonant light scattering in the visible spectral range. Recent research has focused on utilizing this phenomenon to improve the radiative cooling performance of electrospun membranes by introducing such micro/nano particles with a size comparable to the solar wavelength. The particles are usually incorporated into the electrospun fibers by blending them into the spinning solutions. For instance, Jayathilaka et al. [57] studied the radiative cooling performance of electrospun fibers doped with Al_2_O_3_, SiO_2_, and TiO_2_ particles. It was found that, when the fibers doped with Al_2_O_3_ and SiO_2_ particles were stacked together and the final membrane thickness was controlled at 1.1 mm, the membrane could cool as high as ~22.3 °C below the ambient temperature. Jing et al. [63] made an electrospun membrane consisting of polyvinylidene fluoride/alumina (PVDF/Al_2_O_3_) ultrafine fibers having a diameter ranging from 0.5 μm to 2.5 μm for daytime radiative cooling below the ambient temperature, based on the high emissivity of PVDF in the transparent atmospheric window, strong sunlight reflectivity of Al_2_O_3_ nanoparticles, as well as the scattering of solar light by fibers that have diameter equivalent to the wavelength of the solar light. The membrane shows an atmospheric window emissivity of 0.95, a solar reflectivity of 0.97, a net radiative cooling power of 82.7 W/m^2^, and a temperature drop of about 4.0 °C under direct sunlight. Xiao et al. [56] fabricated an infrared-radiation-enhanced nanofiber membrane (NFM) composed of polyamide 6 (PA6) nanofibers and randomly distributed SiO_2_ submicron spheres. Under a clear sky, the NFM membrane results in temperatures that are about 0.4–1.7 °C lower than those of commercial textiles when they are covered by dry or wet hands, and temperatures that are about 1.0–2.5 °C lower than the ambient temperature when tested in a closed device with thermal conduction and convection being isolated. For an electrospun membrane composed of PVDF electrospun fibers and randomly distributed SiO_2_ microspheres [65], an average solar reflectivity of 92.3% and an average transmittance of 0.86 in the atmospheric window were obtained. When the membrane was irradiated under sunlight (700.8 W/m^2^), its temperature was 5.8 °C lower than the ambient temperature. Another electrospun membrane consisting of PVDF-HFP fibers incorporated with SiO_2_ nanoparticles shows a reflectivity exceeding 0.97 in the solar band and emissivity over 0.94 in the atmospheric window, leading to a cooling effect of 15.9 °C under direct sunlight [104].

In addition to the solution blending method, particles can be combined into the electrospun membranes by post-treatment. For instance, Wang et al. [64] prepared PVDF/tetraethyl orthosilicate (TEOS) electrospun membrane with numerous nanopores in the fibers resulted from the solvent-evaporation-induced phase separation, and then SiO_2_ microspheres (diameter 6–14 μm) were introduced into the membrane by simple emulsion deposition. The composite membrane shows an average IR emissivity of higher than 0.96, a solar light reflectivity of ~97%, an average cooling power of 61 W/m^2^, and a temperature drop of 6 °C under direct sunlight radiation (with a peak solar intensity of 1000 W/m^2^). Hu et al. [59] prepared polyvinylidene fluoride–hexafluoropropylene (PVDF-HFP) electrospun membrane, followed by the electrospraying of SiO_2_ nanoparticles, to realize a high solar reflectivity of 98.5% and an average mid-infrared emissivity of higher than 95%. Under sunlight, the average temperature decrease of the membrane was 11.6 °C compared with the surrounding air. Similarly, Meng et al. [105] prepared bead (SiO_2_ nanoparticles)-on-string (PVDF-HFP fibers) structured membrane by electrospinning and electrostatic spraying. The membrane achieves average solar reflectance of 97.8% and average atmospheric window emittance of 96.6%, leading to sub-ambient temperature drops of 11.5 and 4.1 °C in daytime and nighttime outdoor conditions, respectively.

### 5.3. Designing Multilayer Structures

**Figure 5:**
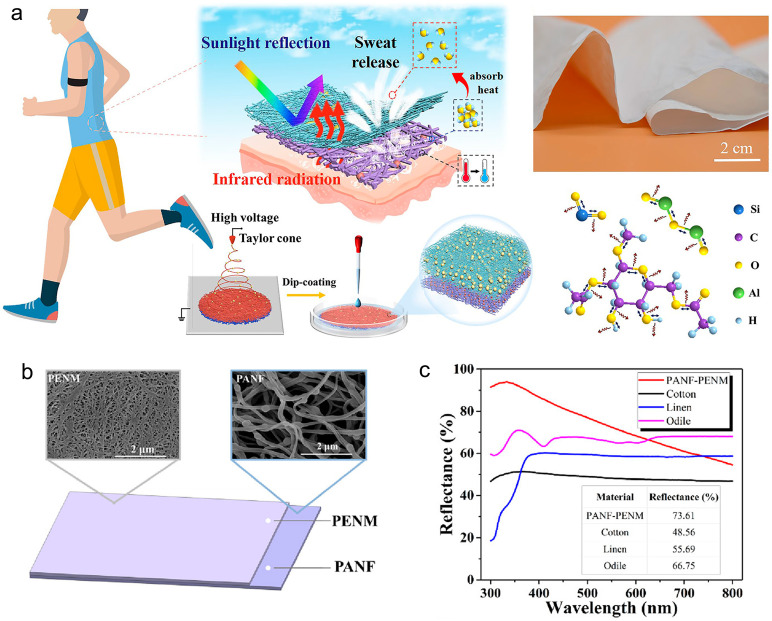
(**a**) Schematic illustration of the preparation process of the bilayer membrane, its photograph, and the schematic illustration of the IR emission by bond vibration of CA, Al_2_O_3_, and SiO_2_. Reprinted with permission from [53]. Copyright 2022 American Chemical Society. (**b**) Structural design and SEM images of the PANF-PENM membrane. Reprinted with permission from [51]. Copyright 2018 American Chemical Society. (**c**) Reflectance curves of PANF-PENM membrane and other commercial textiles. Reprinted with permission from [51]. Copyright 2018 American Chemical Society.

By incorporating different materials with complementary properties, the resulting radiative cooling material can not only improve its cooling performance, but also compensate for any shortcomings associated with a single material. The multilayer structure design strategy has led to the improvement of radiative cooling and also improved the compatibility of radiative cooling function with practical performance requirements such as moisture permeation and waterproofness. For instance, Zhang et al. [53] prepared an electrospun membrane composed of a bottom CA/Al_2_O_3_ layer (near the skin) and a top PA6/SiO_2_ layer nanofiber membrane (Figure 5a). For the top layer, the pore size (300–500 nm) and fiber diameter (100 nm) make it reflect in the ultraviolet-visible region, and the addition of SiO_2_ increases its reflectivity in the near-infrared region. In the bottom layer, the pore size of 900–1500 nm and the addition of Al_2_O_3_ further improve the reflectivity in the near-infrared region. Regarding the IR radiative cooling, the CA in the bottom layer shows a desirable selective emissivity within the atmospheric window, while in the top layer, the PA6 is an IR-transparent material, and it allows thermal radiation emitted by the bottom CA/Al_2_O_3_ layer to transmit effectively. Overall, the whole membrane achieves a high reflectivity of 99.16% in the 0.3–0.76 μm wavelength range and 88.60% in the 0.76–2.5 μm wavelength range, as well as a selective emissivity of 78.13% in the 8–13 μm window.

In addition to the combination of two or more layers of electrospun membranes, an additional layer that is not prepared by electrospinning can also be used to enhance the cooling performance. For instance, Song et al. [51] used nanoporous PE membrane (PENM, pore size 100–1000 nm) to form a bilayer membrane together with nylon 6 electrospun membrane (PANF) (Figure 5b). The nanoporous PE membrane was used as the outer layer, as it has good IR transmittance and its abundant nanopores results in a strong reflection to visible light (380–760 nm) (Figure 5c). In addition, they also proposed a three-layer film composed of a middle layer of Si_3_N_4_@PVDF electrospun fibers, an outer layer of nanoporous PE membrane, and an inner layer of hydrophilic-modified PE membrane (mPE) [52]. The middle Si_3_N_4_@PVDF layer serves to emit IR, because PVDF has strong absorptions at 8.1, 8.5, 9.3, 11.4, and 11.9 μm, and the major absorption of Si_3_N_4_ is at 10.0 μm. The middle layer also functions to reflect visible light (PVDF fiber diameter 200–600 nm, Si_3_N_4_ particles 200–1000 nm). The outer and inner PE layers further enhance the reflectance to visible light. The purpose of hydrophilic modification of inner PE layer is to facilitate the moisture permeation. This design results in an IR emittance of 87.31% and a sunlight reflectance of 93.28%. Under direct sunlight radiation, the three-layer film shows a high cooling performance, in which the temperature of the skin is 7.7–10.8 °C lower compared to common textiles.

Moreover, Song et al. [28] further combined nanoporous PE membrane with electrospun nylon (PA) and PVDF membranes to form a three-layer film (PA/PVDF/PE). The composite film can induce a temperature drop of 4.5–6.5 °C for the human body under direct sunlight radiation. Besides the nanoporous PE membrane, porous PTFE was also used to compensate for PVDF/SiO_2_ electrospun membrane which has a relatively low reflectance to UV light [54]. Under a clear sky, the PVDF/SiO_2_-PTFE bilayer film achieves radiative cooling performance of ~9.2 °C temperature drop compared to the bare simulated human skin.

Compared to the approach of optimizing the geometric parameters, the methods of incorporating highly reflective nanoparticles and designing multi-layer structures require more intricate fabrication processes, which inevitably increase the cost and complexity of producing the membrane. Despite the added expense and complexity, research results in the literature consistently demonstrate that these methods can provide higher cooling efficiency than single-material or single-layer approaches. Therefore, for applications that require high-performance cooling capabilities, the benefits of these advanced techniques may justify the additional cost and complexity involved in their implementation.

## 6. Dual-Mode Temperature Regulation

Besides the high reflectivity that is often utilized to enhance the radiative cooling performance, additional heating functions can be added without affecting the radiative performance so as to allow radiative cooling membranes to adapt to a wider range of temperature conditions, enhancing their versatility and usefulness in various applications. For instance, Xiang et al. [61] reported an electrospun membrane with dual-functional radiative cooling and solar heating abilities for daytime thermal management. PVDF-HFP electrospun fibers were employed for radiative cooling purpose, and polypyrrole (PPy) was spray-coated onto one side of the PVDF-HFP fibrous membrane for heating purposes. During hot sunny days, the side without PPy (white) can face towards the sun to realize a sub-ambient cooling effect with temperature drop of ~4.5 °C. On the contrary, during cold sunny days, the side with PPy (black) can be turned towards the sun to absorb the sunlight and thus to get a heating temperature of ~35.8 °C (with solar intensity of ~850 W/m^2^).

In another example, a Janus membrane with dual functions of cooling and heating was prepared by consequently spray-coating ZnO nanosheet, carbon nanotube (CNT), silver nanowire (AgNW), and PDMS membrane on the top of the PVDF electrospun membrane [106]. For the cooling mode, the dual-functional membrane exhibited a radiative emissivity of 89.2% and a very low sunlight absorptivity of only 9.4%, and as a result, temperature drops of 8.2–12.6, 9.0–14.0, and 10.9 °C were realized for a substrate, a closed space, and a semi closed space, respectively. However, for the heating mode, a low thermal radiation emissivity of 10.5% and a high sunlight absorptivity of 74.1% were achieved. For a substrate, a closed space, and a semi closed space, the temperature rise was 3.8–4.6, 4.0–4.8, and 12.5 °C, respectively.

To achieve the solar heating function, the above two examples coated additional sunlight-absorbing materials on one side of the electrospun membranes, and it is required to manually flip the membranes for obtaining the cooling-heating switch. Very recently, Li et al. [107] utilized the changes in the optical reflectivity induced by stretching to realize the reversible switching between radiative cooling and solar heating for electrospun polyurethane membrane, where the inherent nature of electrospun membranes plays a critical role. In the radiative cooling mode (0% strain), the membrane shows a high and angular-independent reflectance of 95.6% in the 0.25–2.5 μm wavelength range, an IR emissivity of 93.3% in the 8–13 μm atmospheric window, a temperature drop of 10 °C at midday, and a corresponding cooling power of 118.25 W/m^2^. Under an elongation strain of 80%, the membrane is in the solar heating mode, and it shows a reflectivity of 61.1%, resulting in a net temperature increase of 9.5 °C above ambient of an absorbing substrate and an equivalent power of 220.34 W/m^2^.

Compared with other membranes with single temperature regulation mode and limited temperature regulation range, dual-mode membranes prepared by electrospinning have great potential in practical applications. The dual-mode temperature regulation characteristic can prolong the buffer time for objects in the face of huge and abrupt weather changes, so as to make them more suitable for diverse and complex environments.

## 7. Conclusions and Perspectives

In conclusion, we introduced the basic principles of radiative cooling, the radiative cooling concept of electrospun membranes, and the selection criteria for materials, and summarized the recent advancements in the structural design of electrospun membranes for improved cooling performance, as well as the dual-mode temperature regulation that aims to adapt to a wider range of temperature conditions. Based on the studies reviewed, it can be concluded that the morphology and composition of electrospun membranes can be tailored to optimize their radiative cooling performance, making them a promising candidate for sustainable and energy-efficient radiative cooling technologies. The optimization of geometric parameters, incorporation of highly reflective nanoparticles, and designing multilayer structures have all been effective strategies for enhancing their cooling performance. Moreover, the development of dual-mode temperature regulation has expanded the applicability of electrospun membranes for cooling in a wider range of temperature conditions. Within the scope of this review, the following perspectives were attained:

(1) Based on the diversity of material selection and structural design, it is important to carefully analyze the intended use of the radiative cooling electrospun membranes in order to optimize the material and structure for maximum effectiveness. This will allow us to create radiative cooling electrospun membranes that are compatible with a wide range of applications and offer excellent cooling performance, helping to meet the growing demand for sustainable and energy-efficient solutions.
(2) For accurately comparing the cooling performance of different electrospun membranes, it is very important to standardize the self-built test platform used in the testing process. This involves using the same equipment and providing clear information on the thickness of the membranes being tested, under identical environmental conditions whenever possible. In addition to these standardization measures, it is also important to consider various factors when explaining the cooling performance of a membrane. These factors include the cooling power, temperature difference, sunlight reflectivity, and infrared emissivity. By combining and analyzing these metrics, a more comprehensive and accurate understanding of a membrane’s cooling performance can be obtained.
(3) The cooling effect of electrospun membrane is an important factor to consider, but it should not be the sole focus at the expense of other practical properties. Breathability, wear-resistance, skin-friendliness, mechanical strength, and durability are also crucial characteristics that cannot be overlooked. Only when radiative cooling membranes are developed to be compatible with a variety of practical properties can they truly have value for practical application and successful commercialization in the market. Therefore, it is important to strike a balance between cooling and other practical properties in order to produce high-quality and functional electrospun membranes.

Overall, electrospun membranes are advantageous in their versatility in terms of their composition and structure. They show promise as a material for radiative cooling applications. However, there are also challenges associated with the use of electrospun membranes for radiative cooling. One of the main challenges is their mechanical durability. The membranes can be susceptible to abrasion, stretching, and other types of damage. Additionally, the fabrication process for electrospun membranes can be complex and expensive, which can limit their scalability for large-scale applications. Further research is needed to address these challenges, particularly regarding the improvement of their mechanical durability and scalability. In addition, enhancing their radiative cooling performance is also necessary to fully harness their potential for this application.

## Data Availability

Not applicable.
